# Effect of Antipsychotic Treatment on Neutrophil-to-Lymphocyte Ratio during Hospitalization for Acute Psychosis in the Course of Schizophrenia—A Cross-Sectional Retrospective Study

**DOI:** 10.3390/jcm11010232

**Published:** 2021-12-31

**Authors:** Bartosz Dawidowski, Grzegorz Grelecki, Adam Biłgorajski, Piotr Podwalski, Błażej Misiak, Jerzy Samochowiec

**Affiliations:** 1 Department of Psychiatry, Pomeranian Medical University, 71-460 Szczecin, Poland; aravial.mg@gmail.com (B.D.); grzegorz271195@wp.pl (G.G.); bilgorajski@gmail.com (A.B.); samoj@pum.edu.pl (J.S.); 2 Department of Psychiatry, Division of Consultation Psychiatry and Neuroscience, Wroclaw Medical University, 50-367 Wroclaw, Poland; blazej.misiak@umed.wroc.pl

**Keywords:** neutrophil-to-lymphocyte ratio, antipsychotics, schizophrenia, hypothyroidism, inflammatory markers

## Abstract

Background: Studies have shown that there are deviations in the results of peripheral blood counts, which lead to increased values of the neutrophils-to-lymphocytes ratio (NLR) in schizophrenia. Antipsychotic drugs have proven to lower the levels of pro-inflammatory cytokines and a growing number of studies indicate a similar effect on NLR values. Methods: We identified inpatients with schizophrenia and collected data of NLR at the beginning (NLR_1_) and end (NLR_2_) of hospitalization, the status of antipsychotic medication on admission and potential confounding factors. In the statistical analysis, we applied a linear mixed model. Results: After the inclusion and exclusion process the records of 40 patients (n_p_ = 40) and 71 hospitalizations (n_h_ = 71) were analyzed. We found that in the group of antipsychotics-naive patients, the NLR_1_ were significantly higher than the NLR_2_ values. Such a difference did not occur in the case of non-antipsychotics-naïve patients. Age and the diagnosis of hypothyroidism influenced the value of change in NLR from the beginning to the end of hospitalization in a given patient (ΔNLR). Conclusions: The study confirmed the lowering effect of antipsychotics on NLR values in psychosis. The NLR may potentially be a tool for assessing response to treatment with antipsychotics.

## 1. Introduction

Schizophrenia is a chronic mental illness characterized by positive symptoms (e.g., delusions, hallucinations), negative symptoms (e.g., anhedonia, avolition), and cognitive impairment (e.g., impairment of abstract thinking or executive functions) accompanied by degenerative changes in the nervous tissue of the central nervous system (CNS) [1]. Disturbances in neurotransmission (e.g., dopaminergic or glutamatergic pathways) and nervous tissue metabolism (e.g., in the kynurenine pathway, glucose metabolism, antioxidants metabolism) are also important for the symptomatology of schizophrenia and its etiopathogenesis [2,3,4,5]. Several different hypotheses have been proposed to explain the causes of these disorders, however, growing evidence suggests that immune dysfunction, neuroinflammation, and the associated oxidative stress, additionally modulated by dysregulation of the hypothalamic-pituitary-adrenal axis (HPA axis), may play a key role in the etiopathogenesis of schizophrenia [6,7].

Cytokines are immune system signaling proteins produced by a wide variety of cells, including lymphocytes, macrophages, and granulocytes, which are growth and proliferation factors for various leukocyte fractions [8]. Disturbances in the cytokine network in schizophrenia, both in the blood and in the cerebrospinal fluid, with a distinct imbalance between pro-inflammatory and anti-inflammatory cytokines, are well documented [9,10,11]. They are mainly expressed in elevated peripheral levels of pro-inflammatory cytokines, such as interleukin-1β (IL-1β), interleukin-6 (IL-6), or tumor necrosis factor (TNF-α) [9,11]. These cytokines, apart from causing excessive activation of astrocytes and microglia, probably also influence hematopoiesis and differentiation of cells of the immune system, not directly related to the pathophysiology of schizophrenia. [7,12,13]. Moreover, after treatment of acute psychosis with antipsychotics, the peripheral levels of pro-inflammatory cytokines decrease, suggesting that these drugs may reduce the severity of inflammation and potentially also affect hematopoiesis and mobilization of immune system cells into the blood [14].

The neutrophils to lymphocytes ratio (NLR) is a simple and easily accessible marker of systemic inflammation obtained by blood count of peripheral blood, whose normal values for healthy people are estimated to be 0.78–3.53, or 0.88–4.0, depending on the population studied [15,16]. NLR is largely independent of age and gender in the healthy adult population, which is a significant advantage over other similar indicators such as monocyte to lymphocyte ratio (MLR) or platelet to lymphocyte ratio (PLR) [17]. However, its values may be elevated, among others, in the metabolic syndrome [18], as a result of smoking [19], in arterial hypertension [20], or hypothyroidism [21], which occur more often in patients with schizophrenia than in the general population [22,23,24].

The meta-analysis by Mazza et al. showed that patients in the state of non-affective psychosis had significantly higher NLR values compared to the healthy controls [25]. In turn, the meta-analysis by Karageorgiou et al. showed that the NLR value in patients with schizophrenia was increased both in the first episode of psychosis and in later episodes [13]. In the same meta-analysis, the value of NLR was positively correlated with the intensity of psychotic symptoms [13], as indicated also by the recent study by Zhou et al. [26,27]. Moreover, the study by Özdin et al. demonstrates that also in the state of remission in schizophrenia, the NLR values are higher than in the control group but lower than during relapse [28]. Furthermore, NLR appears to be elevated in patients with schizophrenia regardless of the presence of metabolic syndrome, laboratory markers such as glycemia, triglyceridemia, or cholesterolemia, and smoking status [17,28]. Additionally, patients medicated with antipsychotics have lower NLR values than drug-naïve patients [26,28].

In this study, we hypothesized that the effect of antipsychotic medication is revealed not only by decreased NLR values in patients who received said treatment before admission but also by decreased NLR values at the end of hospitalization compared to the beginning of hospitalization. In addition, we also proposed that it could be possible to predict the NLR value at the end of hospitalization, when the patient is in complete or partial remission, based on the NLR value on admission. To confirm these hypotheses, we adopted the following aims of the study: (1) determining whether a difference between the NLR values at the beginning and the end of hospitalization due to the psychotic episode existed and whether the status of antipsychotic medication during the month before hospitalization, determined based on the patient’s declaration on admission included in the medical records, influenced said difference; (2) determining the influence of other potential cofounding factors on such difference; (3) determining whether the NLR value at the beginning of hospitalization may be used to predict an NLR change to its end, which could contribute to the future use of the indicator as a marker of remission or response to treatment in schizophrenia, and (4) how likely, cofounding factors frequently present in the population of schizophrenia patients may affect the NLR’s change during hospitalization.

## 2. Materials and Methods

### 2.1. Patients and Study Design

Our study was retrospective. We obtained the data from the archives of the medical records of the Department of Psychiatry of the Pomeranian Medical University in Szczecin. The inclusion criteria for patients were as follows: (1) diagnosed with schizophrenia according to the International Classification of Diseases (ICD-10); (2) physical and psychiatric examination performed by an experienced psychiatrist; (3) hospitalization from 1 January 2015 until 31 July 2020.

Selection bias, which can be defined as systematic differences between baseline characteristics of the groups that are compared, is one of the main weaknesses of observational and retrospective studies, in which the selection of a research sample significantly different from the general population may affect the results obtained [29]. One method of reducing the risk of selection bias is to use randomization [30]. For this reason, in our study, we included only a part of randomly chosen that met the inclusion criteria in the study sample patients (300 files, approximately 50% of all files), and then we excluded hospitalizations from this sample based on the exclusion criteria.

The following exclusion criteria were applied to individual hospitalizations of patients in this group, which were as follows: (1) age < 18 and >65 years; (2) use of psychoactive substances other than alcohol within 1 month prior to admission; (3) present on admission or diagnosed during hospitalization: infectious diseases, autoimmune diseases (other than Hashimoto’s thyroiditis), cardiovascular diseases (other than hypertension), cancer, parasitic diseases, gastrological diseases, diabetes, history of major surgery or head injuries; (4) medication with glucocorticosteroids, their analogs, antibiotics, or cytostatics; (5) BMI > 30; (6) termination of hospitalization by discharge on-demand or discharge without partial or complete remission of symptoms; (7) no peripheral blood counts available at the beginning or end of hospitalization; (8) first blood count performed >5 days after admission, and (9) no data on the variables included in the statistical analysis.

Based on routinely collected medical records, we were not able to explicitly exclude patients who met the criteria of the metabolic syndrome due to the lack of triglyceride and high-density lipoprotein (HDL) concentration tests performed in all patients, as well as the lack of waist circumference measurements. Nevertheless, the exclusion of patients with a BMI > 30 and patients diagnosed with diabetes at least partially reduced the risk of including patients meeting the criteria of the metabolic syndrome in the research sample.

In the case of most patients, we were also unable to determine how many of them were in the first psychotic episode (FEP), therefore we could not stratify the research sample into FEP and chronic patients.

Due to the fact that we did not collect data on earlier hospitalizations (before 1 January 2015) of patients included in the research sample, we did not make comparisons between patients hospitalized many times during the study period and those who were hospitalized once. A patient who was hospitalized once in the analyzed period could even be hospitalized earlier or later many times. For this reason, the information on multiple hospitalizations in the analyzed period is not informative.

Due to the frequent occurrence of hypertension and hypothyroidism in the studied population of patients, we decided not to exclude patients diagnosed with them from the study. We included the potential impact of these diseases on the NLR values in the statistical analysis. On the other hand, we decided to exclude patients with cardiovascular disease and diabetes due to the fact that the number of patients with these diagnoses in the research sample was too small to be included in the statistical analysis.

For all hospitalizations included in the study, information from the physical documentation was coded into the electronic database with the participation of 6 independent persons, which included: the number of neutrophils and lymphocytes in the peripheral blood counts performed at the beginning and at the end of a given hospitalization (based on which the corresponding NLR values were calculated), gender, the status of antipsychotics medication within 1 month prior to hospitalization (A_med_—antipsychotics medication status, antipsychotics-naïve—AN, non-antipsychotics-naïve—Non-AN), smoking status, diagnosis of hypertension, diagnosis of hypothyroidism, age, BMI, the time between the first and last complete peripheral blood count (the duration of therapy measured in days) and the time from admission to the first peripheral blood count (t_lag_, measured in days). In addition, data on antipsychotics used during hospitalization were also collected for descriptive purposes. The change in NLR from the first to the last peripheral blood count (ΔNLR) was then calculated based on the NLR values of the first peripheral blood count (NLR_1_) and the NLR of the last peripheral blood count (NLR_2_).

The staff responsible for performing the laboratory tests was not aware of the study or the clinical status of the patients, and all the peripheral blood counts included in the study were ordered and performed as part of routine therapeutic activities. All laboratory analyzes were performed in one, the same commercial laboratory with a permanent contract with the Department of Psychiatry of the Pomeranian Medical University and using the same analyzer. Thus, the occurrence of a batch effect seems unlikely. Due to its retrospective nature, the study did not require the consent of the Bioethics Committee of the Pomeranian Medical University in Szczecin.

### 2.2. Statistical Analysis

The main objectives of the analysis were: (1) determining whether there was a statistically significant difference between NLR_1_ and NLR_2_ and whether the status of A_med_ moderated this difference; (2) determining the influence of other potential confounding factors on the occurrence of such a difference (3) determining whether the NLR_1_ value allows ΔNLR prediction; (4) evaluation of the influence of potential confounding factors on ΔNLR.

Descriptive statistics included standard deviation (SD), median, and mean for continuous variables, as well as frequencies and percentage of all included hospitalizations for categorical variables. For gender, the frequency and percentage were also calculated for all patients included in the study. Due to the fact that not all observations were independent of each other (there were multiple hospitalizations for the same patient), we used a linear mixed model (LMM) for statistical analysis.

LMM is a family of linear models, one of the main applications of which is the analysis of data with repeatable and interdependent measurements (in our study, these are hospitalizations) within the same object (in our study, it is a patient), disregarding the assumption of the independence of observations that applies to classical linear regression models [31]. The use of LMM in the analysis of our data allows one to prevent the impact of multiple hospitalizations of the same patients on the quality of the results obtained. Moreover, LMMs can also be used to include corrections for the possible occurrence of a batch effect, although this was not the immediate goal of our analysis.

To determine the differences between NLR_1_ and NLR_2_, the data were transformed so that the NLR values, regardless of whether the measurement was performed at the beginning or at the end of hospitalization, were represented by the same dependent variable (NLR_x_). On the other hand, the timing of blood count was represented by the categorical grouping variable (2 levels: NLR_1_ and NLR_2_) as a fixed effect that interacted with the Amed categorical variable to control for the effect of pre-hospitalization antipsychotic medication. In addition, the individual patient ID (ID_p_) with the nested hospitalization ID (ID_h_) in it was included in the model as a random effect, so that the dependence of data from different hospitalizations of the same patient did not affect the results, and at the same time to take into account the relationship between the values of NLR_1_ and NLR_2_ in the same hospitalization.

To determine the predictability of ΔNLR with the use of the NLR_1_, a model in which the dependent variable was ΔNLR was fitted. ID_p_ were entered into the model as a random effect, to account for the relationship of data from different hospitalizations of the same patients. NLR_1_ was introduced as the main fixed effect in the model.

In both models, categorical variables were introduced as additional fixed effects, i.e., gender, A_med_, smoking status, diagnosis of arterial hypertension, diagnosis of hypothyroidism, and continuous variables such as age, BMI, duration of therapy, and t_lag_.

In the case of both models, all of the above-mentioned predictors were initially taken into account, and then those that did not significantly improve the goodness of fit of the model were eliminated stepwise from the model. The goodness of fit of the model was assessed based on the Akaike Information Criterion (AIC), treating the predictor as irrelevant if its removal from the model did not increase AIC by >2. The predictors whose elimination from the model caused the smallest increase in AIC were eliminated in the first place. If the difference in AIC values between the models was less than 2, the model with a smaller number of predictors was selected. In the case of the NLR_x_ model, before the predictor elimination, its effect after adjusting for interaction with the grouping variable was also tested to determine its different potential effects on the values of NLR_1_ or NLR_2_ and AN or non-AN patients. Thus, the final models only included predictors that improved their goodness of fit. Such a model-fitting algorithm makes it possible to reduce the risk of overfitting the model, as well as to obtain results that do not reflect real dependencies.

During the stepwise elimination of predictors and the assessment of goodness of fit, restricted maximal likelihood (REML) was not used to obtain the correct AIC values. Instead, the maximum likelihood (ML) was used. In further stages of the analysis, REML was used to more accurately assess the values of the coefficients and confidence intervals (CIs).

Then, the possible interactions between the predictors that were included in the final model for ΔNLR were considered, using a similar methodology initially introducing all of them and interactions between them into the model and looking for the model best suited to the data based on AIC. When the difference in AIC values between the models was less than 2, the model with fewer predictors and interactions was selected.

The linearity of the predictors was checked by visual assessment of the plot of the predictions versus residual plots. The homogeneity of variance was checked by analysis of variance (ANOVA) of the linear model with the squared absolute residual values as the dependent variable and IDp (in the ΔNLR model) or ID_p_ with nested ID_h_ (in the NLR_x_ model) as predictors. The independence of the residuals was checked by visual assessment of the residuals versus predictors plots. In turn, the normality of the residual distribution was checked by visual assessment of the Quartile-Quartile plot (Q-Q plot).

In the case of the model for NLR_x_, the distribution of residuals significantly differed from the normal distribution, therefore a logarithmic transformation of the dependent variable was performed, which further improved the goodness of fit of this model to the data.

Post-hoc tests were performed using the Kenward–Roger method to evaluate the differences between the groups in the model for log(NLR_x_).

We used 95% confidence intervals (95% CI) to assess the statistical significance of the predictors. Additionally, although the methodological correctness of the *p*-value application for linear models with mixed effect is still not unequivocal, we calculated them using the Satterthwaite *t*-tests.

W used the conditional _pseudo_R^2^ (_pseudo_R^2^_c_) calculated by the Nakagawa method, which determines the proportion of variance explained by the entire model taking into account the fixed and random effects, as well as the marginal _pseudo_R^2^ (_pseudo_R^2^_m_), which determines the proportion of variance explained by the fixed effects of the model.

All *p* values used in the analysis were two-tailed. The significance level was α = 0.05. All stages of the statistical analysis were performed in R studio version 4.0.3 using the lmer, lmerTest, car, and MuMln packages.

## 3. Results

### 3.1. Patients Characteristics

**Figure 1:**
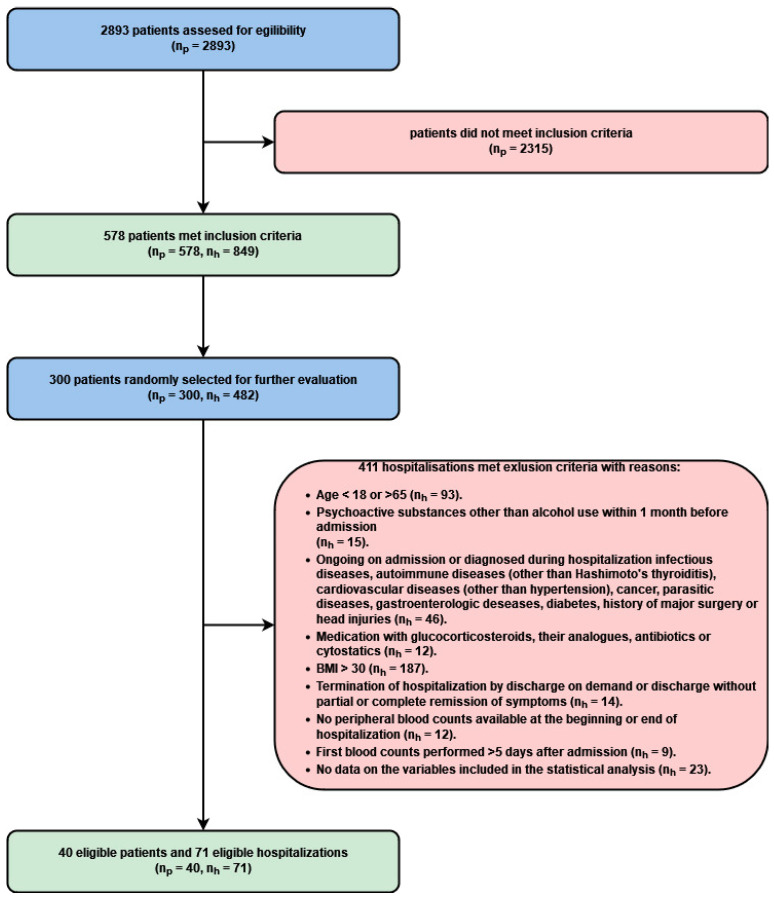
The process of inclusion and exclusion from the study. n_p_ = number of patients, n_h_ = number of hospitalizations.

Inclusion criteria for the study were met by 578 patients with a total of 849 hospitalizations. From this group, 300 patients were randomly selected, with a total of 482 hospitalizations during the study period. After excluding hospitalizations in accordance with the established criteria, the analysis included data on 40 patients (n_p_ = 40) and 71 hospitalizations (n_h_ = 71). The study inclusion and exclusion processes are shown in Figure 1. Descriptive statistics of the research group are presented in Table 1 for continuous variables and categorical variables in Table 2. Among the hospitalizations that were ultimately included in the research sample, in n_h_ = 12 (16.9%), pharmacotherapy with only one antipsychotic was administered, and in n_h_ = 59 (83%), pharmacotherapy with more than one drug from this group was administered. During the time period included in the study, 16 patients (n_p_ = 16, 40%) were hospitalized more than once. The exact counts for each comorbidity can be found in Table S1.

### 3.2. The Difference between the Values of NLR_1_ and NLR_2_

**Figure 2:**
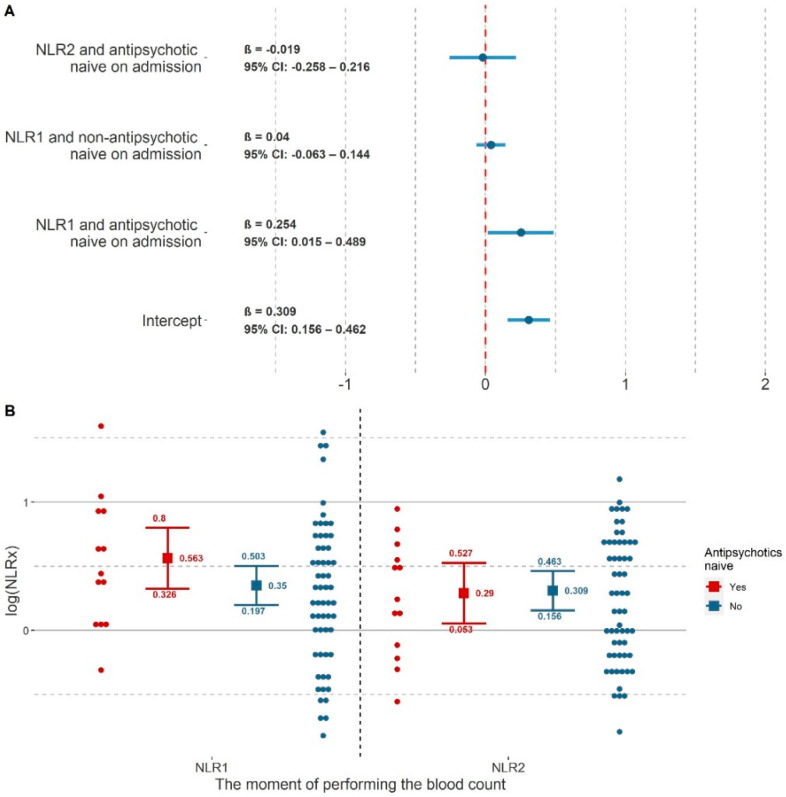
Final model for log(NLR_x_) (**A**) Coefficients plot of the final model for log(NLR_x_). NLR_1_—neutrophil to lymphocyte ratio value from the first peripheral blood count. NLR_2_—neutrophil to lymphocyte ratio value from the last peripheral blood count. (**B**) Visualization of the final model for log(NLR_x_). The squares represent the predicted mean values for each group. 95% confidence intervals were also marked. The colors represent the status of taking antipsychotic drugs during the 1 month prior to admission. Points are individual cases where a shift has been applied for overlapping points.

In the final model, the only fixed effect which remained was the interaction between the grouping variable and A_med_. Stepwise elimination of the remaining predictors and their interactions with the grouping variable showed that they did not improve the goodness of fit of the model. The results of this model are summarized in Table 3 and Figure 2 for fixed effects and Table 4 for random effects. The fixed effects of the model explained a small proportion of the variance (_pseudo_R^2^_c_ = 0.020) as opposed to the random effects (_pseudo_R^2^_m_ = 0.683). The low _pseudo_R^2^_c_ value may be due to the fact that, despite the statistically significant difference between the mean values of NLR_1_ and NLR_2_ in the group of AN patients, only in 5 hospitalizations (n_h_ = 5) the value of the difference between NLR_1_ and NLR_2_ was higher than the differences between the means (Figure S3). The model had insignificantly better goodness of fit than the original model with AIC = 141.275. The model predictors were linear, variance was homogeneous (ID_p_: F(39, 86) = 0.433, *p* = 0.998; ID_h_: F(16, 86) = 0.413, *p* = 0.976), residuals were independent and normally distributed.

The results of the original model with all predictors are summarized in Table S2 and Figure S1 for fixed effects, and Table S3 for random effects. This model had insignificantly lesser goodness of fit to the data than the final model (AIC = 142.709, REML = 165.7). It also explained the greater proportion of NLR_x_ variance (_pseudo_R^2^_c_ = 0.184, _pseudo_R^2^_m_ = 0.729), which was probably due to overfitting the model.

**Figure 3:**
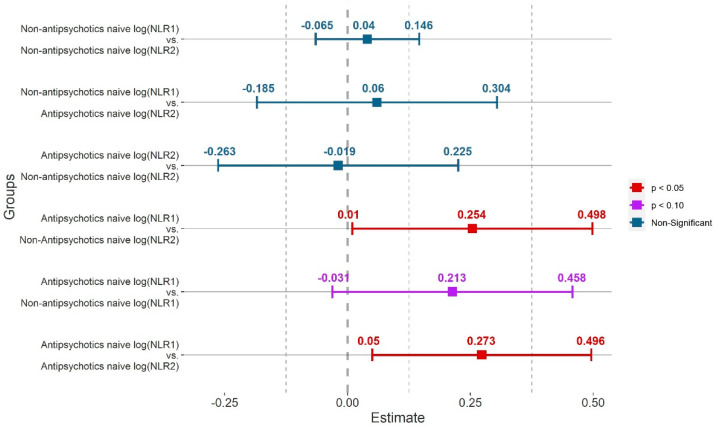
Summary of post-hoc test results for the final model for log (NLR_x_). NLR_1_—neutrophil to lymphocyte ratio value from the first peripheral blood count. NLR_2_—neutrophil to lymphocyte ratio value from the last peripheral blood count. 95% confidence intervals and marginal means have been marked. Red indicates a statistically significant result, violet indicates a statistical trend, and blue indicates statistically insignificant *p* values.

Post-hoc tests showed that AN patients had a significantly higher log(NLR_1_) than log(NLR_2_) (β = 0.273, t = 2.447, *p* = 0.017, 95% CI: 0.050–0.496) and a significantly higher log(NLR_1_) than log(NLR_2_) of non-AN patients (β = 0.254, t = 2.065, *p* = 0.042, 95% CI: 0.010–0.498). The difference between the log(NLR_1_) of non-AN patients and the log(NLR_1_) of AN patients were non-significant, but statistical trend was shown to higher log(NLR_1_) values in the latter group (β = 0.213, t = 1.736, *p* = 0.086, 95% CI: −0.031–0.458). There were no statistically significant differences between log(NLR_1_) in non-AN patients and log(NLR_2_) in AN patients (β = 0.060, t = 0.485, *p* = 0.086, 95% CI: −0.185–0.304), between log(NLR_1_) and log(NLR_2_) in AN patients (β = 0.040, t = 0.765, *p* = 0.447, 95% CI: −0.065–0.146), and also between log(NLR_2_) of non-AN patients and log(NLR_2_) of AN patients (β = −0.019, t = −0.157, *p* = 0.876, 95% CI: −0.263–0.225). A summary of the post-hoc test results is provided in Table 5 and Figure 3.

### 3.3. The Difference between the Values of NLR_1_ and NLR_2_

The results of the primary model (model_P_), including all predictors, are summarized in Table S4 and Figure S2. This model had significantly lesser goodness of fit for the data than the final model (model_F_) (AIC = 109,979, REML = 131.3). It also explained the smaller proportion of the variance ΔNLR (_pseudo_R^2^_c_ = 0.750, _pseudo_R^2^_m_ = 0.619).

**Figure 4:**
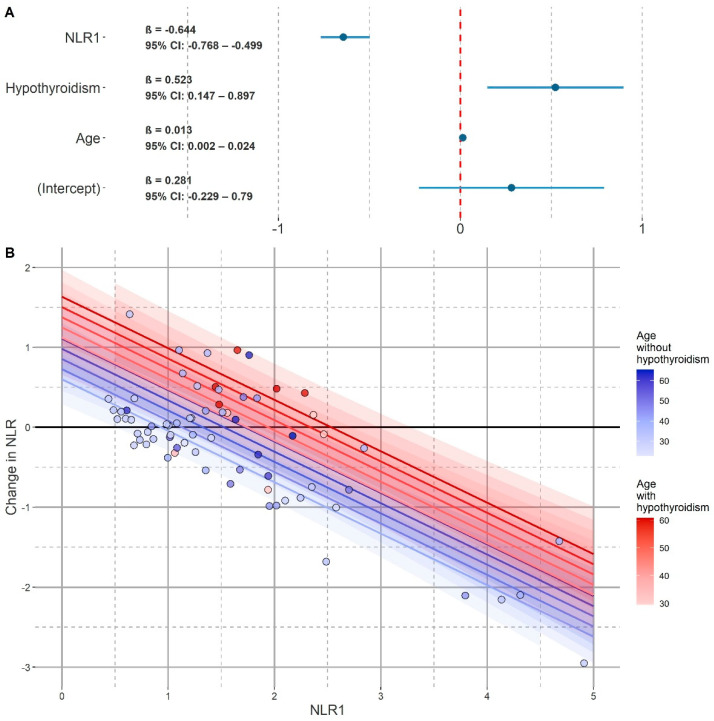
Final model for ΔNLR (model_F_). (**A**) Coefficients plot of the final model for ΔNLR (model_F_). NLR_1_—neutrophil to lymphocyte ratio value from the first peripheral blood count. (**B**) Visualization of model_F_. The horizontal axis represents the value of NLR_1_. The vertical axis represents the change in NLR from peripheral blood counts performed at the beginning of hospitalization to the pre-discharge examination. Patients diagnosed with hypothyroidism were marked on the blue scale, and patients diagnosed with hypothyroidism on the red scale. The darker the shade, the higher the age of the patient. Similarly, different colors of the lines represent predictions for patients without a diagnosis of hypothyroidism (blue scale) and with a diagnosis of hypothyroidism (red scale) and at different ages (the darker the shade, the higher the age). The 95% confidence intervals were marked in a similar manner.

In the final model (model_F_), there were only statistically significant fixed effects shown in Table 6, i.e., age (β = 0.013, t = 2.143, *p* = 0.042, 95% CI: 0.002–0.024), diagnosis of hypothyroidism (β = 0.523, t = 2.695, *p* = 0.012, 95% CI: 0.147–0.897) and NLR_1_ (β = −0.643, t = −9.960, *p* <0.001, 95% CI: −0.768–−0.499). ID_p_ was also taken into account as a random effect (SD_ID_ = 0.303, SD_residuals_ = 0.388). The goodness of fit was not improved by the following predictors: gender, smoking status, hypertension diagnosis, duration of therapy, BMI, A_med_, and t_lag_. These predictors were not included in the model_F_ and it can be assumed that ΔNLR was largely independent of them. The model predictors were linear, variance homogeneous (F(39, 31) = 0.242, *p* = 1.000), the residuals were independent and had a normal distribution. This model fit the data well and had AIC = 101.68 without the use of REML. Using REML (REML = 107), the model had the values of _pseudo_R^2^_c_ = 0.772 and _pseudo_R^2^_m_ = 0.634. The visualization of the model and the graph of its fixed effects coefficients are presented in Figure 4.

**Figure 5:**
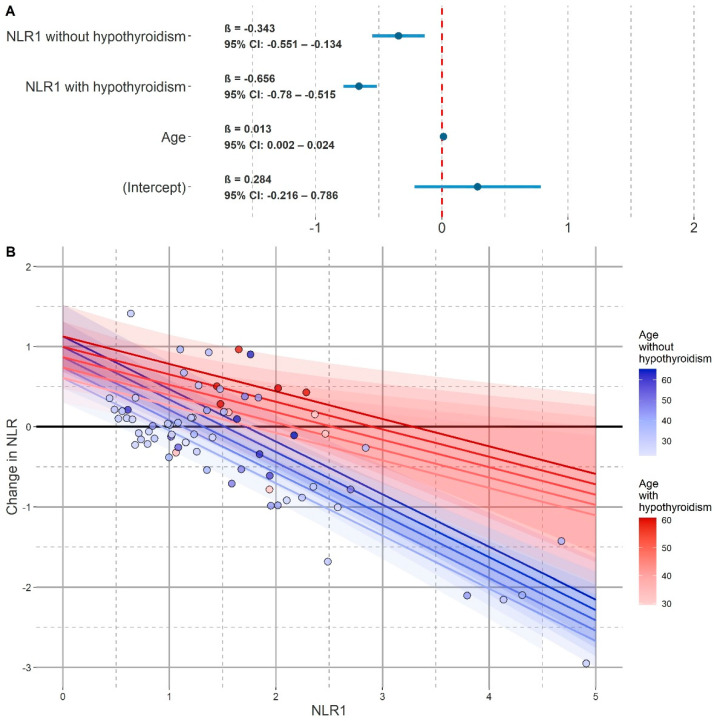
Model for ΔNLR with interaction (model_I_). (**A**) Coefficients plot of model for ΔNLR with interaction (model_I_). NLR_1_—neutrophil to lymphocyte ratio value from the first peripheral blood count. (**B**) Visualization of model_I_. The horizontal axis represents the value of NLR_1_. The vertical axis represents the change in NLR from peripheral blood counts performed at the beginning of hospitalization to the pre-discharge examination. Patients diagnosed with hypothyroidism were marked on the blue scale, and patients diagnosed with hypothyroidism on the red scale. The darker the shade, the higher the age of the patient. Similarly, different colors of the lines represent predictions for patients without a diagnosis of hypothyroidism (blue scale) and with a diagnosis of hypothyroidism (red scale) and at different ages (the darker the shade, the higher the age). The 95% confidence intervals were marked in a similar manner.

We then repeated this procedure for a model including age, diagnosis of hypothyroidism, and NLR_1_, and possible interactions between them. The model with interaction (model_I_) obtained this way was slightly but significantly better fitted to the data (AIC = 98.629). The model calculated using REML (REML = 105.4), in addition to the random effect of the patient’s ID (SD_ID_ = 0.298, SD_residuals_ = 0.379), contained the following fixed effects shown in Table 7: age (β = 0.013, t = 2.230, *p* = 0.035, 95% CI: 0.002–0.024), NLR_1_ in patients without a diagnosis of hypothyroidism (β = −0.656, t = −10.328, *p* <0.001, 95% CI: −0.780–−0.515) and NLR_1_ in patients diagnosed with hypothyroidism (β = −0.343, t = −3.136, *p* = 0.003, 95% CI: −0.551–−0.134). With the introduction of the interaction between NLR_1_ and the diagnosis of hypothyroidism, the variables of hypothyroidism and NLR_1_ no longer improved the goodness of the fit of the model to the data. Interactions between age and NLR_1_ as well as age and hypothyroidism diagnosis also did not improve the goodness of fit of the model. In the case of the interaction model, all LMM assumptions were still met. The model also explained a slightly higher proportion of the ΔNLR variance with the values of _pseudo_R^2^_c_ = 0.783 and _pseudo_R^2^_m_ = 0.650. The visualization of the model and the graph of its fixed effects coefficients are presented in Figure 5.

Table S5 presents a comparison of the goodness of fit statistics of the original, final, and interaction models.

## 4. Discussion

In this retrospective study, we analyzed the effect of antipsychotics on the NLR value in patients hospitalized due to exacerbation of schizophrenia. The NLR values from the first blood count after admission to the hospital (NLR_1_) in patients who were antipsychotic-naïve before admission (AN) were statistically significantly higher than the NLR values from the last blood count (NLR_2_) and the analogous values in patients who were non-antipsychotic-naïve (non-AN). Although the difference between NLR_1_ values in non-AN and AN patients was not statistically significant, we showed a trend towards elevated NLR_1_ values in AN patients versus non-AN patients. The difference between NLR_2_ values in non-AN patients and AN patients was not statistically significant. The obtained results suggest that antipsychotics reduce the NLR values to a similar level both during and before hospitalization, even though both groups of patients were admitted due to an exacerbation. The reported differences were also independent of BMI, duration of therapy, hypertension, hypothyroidism, smoking, gender, time from admission to the first blood count (t_lag_), and age, although the small sizes of the groups made it impossible to take into account the influence of the interaction of the third or a greater degree.

We have also shown that knowing the NLR_1_ value we can predict with high probability the change in the NLR value until the measurement of NLR_2_ when the patient achieves partial or complete remission (ΔNLR). Moreover, we have shown that ΔNLR is independent of BMI, duration of therapy, hypertension diagnosis, smoking status, gender, antipsychotics naivety status on admission (A_med_), and t_lag_. Our study suggests that ΔNLR during hospitalization increases with age, i.e., the possible decrease in NLR value is smaller, and the possible increase is greater in older patients. Additionally, to our knowledge, we were the first to consider the possible influence of hypothyroidism on ΔNLR during hospitalization due to an episode of psychosis in patients diagnosed with schizophrenia. In schizophrenic patients with coexisting hypothyroidism, ΔNLR values were higher than in patients without diagnosed hypothyroidism, thus in such patients, the NLR decreased to a lesser degree or increased more during hospitalization. To put it another way, hypothyroidism interacted with NLR_1_ to reduce the effect of high NLR_1_ values on ΔNLR.

We replicated the results of the meta-analysis by Mazza et al. on the statistical trend to higher values of NLR_1_ in AN patients compared to non-AN patients [25]. Zhou et al. showed significant differences in NLR_1_ values between these groups of patients, taking into account the larger AN sample (however, smaller than in the meta-analysis by Mazza et al.) and the larger non-AN sample [25,26]. Likewise, the meta-analysis by Karageorgiou et al., including the largest sample of AN patients, showed significantly higherNLR_1_ values in AN patients compared to non-AN patients [13]. Similarly, based on a much smaller research sample than ours, the study by Kovacs et al. demonstrated statistically significant differences [27]. Therefore, it is possible that the lack of a significant statistical difference in NLR_1_ values between AN and non-AN patients in our study may result from the smaller size of the research sample, inclusion and exclusion criteria used, or logarithmic transformation of the dependent variable. The significant difference shown in our study between the values of NLR_1_ and NLR_2_ in AN patients with the simultaneous lack of significant differences between these values in non-AN patients further supports this interpretation and is consistent with the results obtained by Bustan et al. on a smaller sample [32].

The independence of the differences between NLR_1_ and NLR_2_ in both AN and non-AN patients on the BMI value seems to be consistent with the results of the studies by Kovacs et al., Semiz et al., and Bustan et al., which showed no influence of this factor on the differences in NLR_1_ values between patients and the healthy control group [27,32,33]. Similarly, in line with our results, the meta-analysis by Karageorgiou et al. and the study by Kovacs et al. showed that smoking had no significant effect on the NLR_1_ values. [13,27]. Our suggested lack of gender influence is also consistent with the results of meta-analyses and individual studies [13,25,27,28]. Although it has been reported that elevated NLR values may be associated with an increased risk of developing arterial hypertension, in our study, in both the ΔNLR and the differences between NLR_1_ and NLR_2_, we did not find any effect of hypertension on these values in the population of patients diagnosed with schizophrenia [20]. In our study, these confounding factors did not have a significant impact on the ΔNLR values, which further supports the observation that the NLR values are largely independent of them, regardless of antipsychotic medication and clinical condition.

The independence of ΔNLR and the values of NLR_1_ and NLR_2_ both in the AN and non-AN groups from the duration of the therapy may indirectly indicate that the NLR values correlate with the clinical state because the patients included in the study were in partial or complete remission at the time of the NLR_2_ measurement. Although the meta-analysis of Karageorgiou et al. did not show a correlation of NLR values with the intensity of symptoms in schizophrenia, later studies that used other methods of assessing the clinical condition of patients, such as those of Zhou et al. and Kovacs et al. seem to strongly indicate this kind of dependency [13,26,27].

The lack of influence of t_lag_ on ΔNLR values and the differences between NLR_1_ and NLR_2_ may indirectly indicate that the effect of antipsychotic treatment on NLR values becomes significantly evident after more than 5 days, which was the upper limit of t_lag_ necessary to account for hospitalization in the study. However, it should be noted that we have not analyzed the results in terms of the exact moment of starting antipsychotic treatment.

Age turned out not to significantly affect the differences between NLR_1_ and NLR_2_ in both AN and non-AN patients, which is consistent with the results of meta-analyses by Mazza et al. and Karageorgiou et al. [13,25], however, it significantly affected ΔNLR. Zhou et al. demonstrated significant collinearity between age and NLR_1_, and although in our study we did not detect significant interactions between NLR_1_ and age, this could be due to a smaller research sample and the inability to account for third-degree interactions [26]. The discrepancy between the impact of age on the difference between NLR_1_ and NLR_2_, and the effect of age on ΔNLR may result from a different statistical methodology of fitting the models for both variables. It is also worth noting that we did not take into account the influence of time from the onset of the disease, which, although, as indicated by Zhou et al. does not seem to have a significant effect on the NLR_1_ values, it could interact with the patient’s age at the time of hospitalization [26]. It is also possible that, while age does not contribute to the difference in mean NLR values between groups, it does contribute to a specific ΔNLR value in individual patients.

Hypothyroidism is a common condition associated with a deficiency of thyroid hormones [34]. It is assumed that in European countries hypothyroidism has autoimmunological underpinnings in the vast majority of cases [35]. This disease is more common in patients with schizophrenia than in the general population [22]. In addition, antipsychotics may contribute to the occurrence of hypothyroidism, possibly both by negatively affecting the activity of the hypothalamic-pituitary-thyroid axis, disturbing iodine metabolism, and inducing the formation of autoantibodies [36]. For these reasons, the influence of hypothyroidism on NLR values, as demonstrated by the Önalan and Dönder study, may be particularly important for its use in clinical practice [21]. In our study, the diagnosis of hypothyroidism did not significantly alter the differences in NLR_1_ and NLR_2_ values in the AN and non-AN groups, but it had a significant impact on ΔNLR. As in the case of age, it may be related to a different methodology for fitting both models to the data or a relatively small proportion of patients diagnosed with hypothyroidism in our research sample. However, the ΔNLR results seem to indicate that although hypothyroidism may not be substantial to the differences between NLR_1_ and NLR_2_ in the general population of patients, it may heavily affect the outcome of antipsychotics in the subpopulation of patients with this comorbidity. Future studies should take into account the fact that in patients with hypothyroidism, the NLR values may not only not decrease, but also, as in the case of most of our patients, increase during hospitalization. Such studies should also control their results in terms of the levels of thyrotropin, thyroid hormones, and anti-thyroid antibodies in the blood due to possible interference of subclinical forms of hypothyroidism.

Medicating with antipsychotics within 1 month prior to admission (A_med_), despite statistically significant influence on differences between NLR_1_ and NLR_2_ values, was non-significant in the case of ΔNLR. It may be related to the use of NLR_1_ values as a predictor in the model for ΔNLR. The statistical trend shown by us in the differences between the NLR_1_ values between AN and non-AN patients indicates that these values are likely to be dependent on A_med_. The lower _pseudo_R^2^_c_ value for the log(NLR_x_) model compared to the ΔNLR model may also be related to the fact that despite the statistically significant difference between the mean values of NLR_1_ and NLR_2_ in the AN group, only in a small part of hospitalizations (n_h_ = 5) the difference between NLR_1_ and NLR_2_ was higher than the difference between the averages (Figure S3). As shown by the model for ΔNLR, the NLR value decreased during hospitalization more significantly in patients with a higher baseline NLR value (NLR_1_), which, however, does not exclude the influence of pharmacotherapy with antipsychotics on the NLR value, among other things, because these drugs can only lower the NLR value to a certain baseline level. For this reason, NLR_1_ values alone would be a much better predictor of NLR_2_ values, which is further supported by a much higher proportion of explained ΔNLR variance in models incorporating NLR_1_ as a predictor compared to the model for NLR_x_. This could explain both the lack of significance of A_med_ in the case of the model for ΔNLR and indicate the greater potential usefulness of using ΔNLR as a marker of response to pharmacotherapy with antipsychotics than the usefulness of NLR values alone.

There is ample evidence from meta-analyses of alterations in the cytokine system in patients with schizophrenia [9,10,11] and the effect of antipsychotic drugs on their levels peripherally [14]. The levels of some cytokines peripherally also correlate with the intensity of schizophrenia symptoms [37,38]. One of the key pro-inflammatory cytokines whose blood levels are elevated in both psychotic and remitted patients, compared to the healthy controls as well as lowered by antipsychotic drugs, is interleukin-6 (IL-6) [9]. Likewise, the levels of interferon-γ (IFN-γ) in the peripheral blood are elevated both in the first and subsequent episodes of psychosis, but unchanged or even lower compared to the healthy controls during remission [9,37]. Similarly, IFN-γ levels are lowered by antipsychotic drugs [14,39]. Not as apparent but similar effects of antipsychotic drugs may also apply to other cytokines such as interleukin-1β (IL-1β), interleukin-4 (IL-4), or tumor necrosis factor α (TNF-α) [9,11]. One of the important common features of IL-1β, IL-6, IFN-γ and TNF-α is their effect on hematopoiesis, in particular stimulation of the differentiation, maturation, and proliferation of cells of the myeloid lineage, which includes neutrophils, and not lymphoid lineage [40]. It is, therefore, possible that elevated levels of these cytokines in schizophrenic patients may increase the number of neutrophils in the blood, but do not significantly affect the number of lymphocytes. Such activity could be associated with increased NLR values in the period of and would be consistent with the reports on the increased number of neutrophils in the blood in patients diagnosed with schizophrenia, while the number of lymphocytes in the blood of this group of patients was within the normal range [41]. At the same time, the decreasing levels of these cytokines, especially IFN-γ, due to the action of antipsychotic drugs, could reduce the NLR presented by the results of our study. However, the confirmation of such a cause-and-effect sequence requires further future research.

Our study has certain limitations. First of all, it was a retrospective study, which made it impossible to fully control the results obtained by us in terms of the patients’ clinical condition. We did not use data obtained through more quantifiable methods of its assessment, such as scales, inventories, or structured interviews. All the premises relating to this were indirect. Moreover, we did not have data on the age of onset of the disease and its course before hospitalizations included in the study. We also did not control the results obtained by us in terms of the use of specific forms of pharmacotherapy or other methods of treatment. We also did not take into account the levels of thyroid hormones, thyrotropin, and anti-thyroid antibodies, which would allow us to capture the impact of subclinical hypothyroidism. Likewise, we did not collect data on other markers of inflammation, such as blood C-reactive protein or cytokine levels, which made it impossible to assess the independence of NLR as a marker of treatment response. The same problem applies to the lack of complete diagnosis of metabolic syndrome in patients included in the research sample. The meta-analysis by Mazza et al. suggested that NLR may be a better marker for FEP patients [25]. Unfortunately, because we did not perform the stratification of chronic and FEP patients, we were unable to address this thesis. Finally, we based our study on a relatively small research sample, which limits the possibility of making more certain conclusions about the variability of NLR values during hospitalization due to exacerbation of schizophrenia. A small research sample also made it impossible to thoroughly investigate possible interactions between cofounders during statistical analysis, which could potentially prevent the capture of the influence of individual cofounders on NLR values. Although the mean values of NLR_1_ and NLR_2_ in the group of AN patients were statistically significantly different, in the case of the majority of specific hospitalizations, the differences between the values of NLR_1_ and NLR_2_ were not greater than the difference between the mean values of NLR_1_ and NLR_2_ in this group. However, the statistical significance of the results obtained by us, combined with the lower risk of selection bias due to randomization, does not seem to indicate that the statistical power was too low to perform the analyzes.

## 5. Conclusions

In conclusion, in our retrospective study, we showed that NLR values have been significantly different at the beginning and the end of hospitalization in patients who had not taken antipsychotic drugs within one month before admission to the hospital due to exacerbation of schizophrenia. We also showed no significant differences between such NLR values in patients who had been treated with antipsychotics before admission and a statistical trend for differences between the NLR values on admission between patients treated with antipsychotics on admission and antipsychotics-naïve patients. Eventually, we also indicated the predictive potential of NLR at admission versus discharge NLR after partial or complete remission. Such an approach could discount the effects of previous antipsychotic medication but would require consideration of age and the diagnosis of hypothyroidism. The assessment of the change in NLR with the use of antipsychotics could potentially be used to assess the response to pharmacotherapy in patients with schizophrenia.

## Figures and Tables

**Table 1:** Descriptive statistics for continuous variables were included in the study.

Predictor	Median	Mean	SD
Age (years)	35.953	39.510	11.500
BMI	24.802	24.398	3.233
t_lag_ (days)	2.000	2.746	1.779
Duration of therapy (days)	33.000	40.694	20.767
NLR_1_	1.369	1.614	0.974
NLR_2_	1.274	1.421	0.666
ΔNLR	−0.090	−0.193	0.767

SD—standard deviation, BMI—Body-Mass Index, t_lag_—time from admission to the first blood count, NLR_1_—neutrophil to lymphocyte ratio value from the first peripheral blood count, NLR_2_—neutrophil to lymphocyte ratio value from the last peripheral blood count, ΔNLR—change in the value of the neutrophil to lymphocyte from the first to the last peripheral blood count.

**Table 2:** The frequencies of the categorical variables included in the study and the number of hospitalizations during which the patient was taking the given antipsychotic drug.

Variable	All Hospitalizations (n_h_ = 71)	All Patients (n_p_ = 40)
Male	–	21 (52.5%)
Non-antipsychotics naïve	58 (81.7%)	34 (85.0%)
Smoking	39 (54.9%)	21 (52.5%)
Hypertension	12 (16.9%)	4 (10%)
Hypothyroidism	12 (14.1%)	6 (15%)
Amisulpiride	9 (12.7%)	8 (20%)
Aripiprazole	16 (22.5%)	14 (35%)
Chloroprotixen	3 (4.2%)	3 (7.5%)
Flupentixol	4 (5.6%)	3 (7.5%)
Haloperidol	2 (2.8%)	2 (5%)
Clozapine	30 (42.3%)	13 (32.5%)
Quetiapine	7 (9.9%)	6 (15%)
Levomepromazine	7 (9.9%)	6 (15%)
Olanzapine	28 (39.4%)	22 (55%)
Perazine	6 (8.5%)	4 (10%)
Promazine	27 (38%)	18 (45%)
Risperidone	12 (16.9%)	11 (27%)
Sulpiride	8 (11.3%)	6 (15%)
Zuclopentixol	13 (18.3%)	5 (12.5%)
Valproate	30 (42.3%)	18 (45%)
Lamotrigine	12 (17.0%)	9 (22.5%)
Lorazepam	22 (31.0%)	12 (30%)
Clonazepam	6 (8.5%)	4 (10%)
Diazepam	2 (2.8%)	1 (2.5%)
Estazolam	7 (9.9%)	4 (10%)
Lithium	2 (2.8%)	1 (2.5%)
Trazodone	1 (1.4%)	1 (2.5%)
Sertraline	2 (2.8%)	1 (2.5%)
Fluoxetine	2 (2.8%)	1 (2.5%)
Zolpidem	3 (4.2%)	1 (2.5%)
Escitalopram	1 (1.4%)	1 (2.5%)
Pregabalin	2 (2.8%)	1 (2.5%)

The percentage in the whole group is given in parentheses. n_p_—number of patients, n_h_—number of hospitalizations. The column for all patients includes the occurrence of a given factor if it appeared in any of the hospitalizations of a given patient.

**Table 3:** Summary of the fixed effects of the final model for log(NLR_x_). NLR_1_—neutrophil to lymphocyte ratio value from the first peripheral blood count, non-AN—non-antipsychotics-naïve, AN—antipsychotics-naïve. 95% CI—95% confidence interval. Statistically significant *p*-values are **bolded**.

Fixed Effect	β	t	*p*	95% CI
Intercept	0.309	3.960	**<0.001**	0.156–0.462
NLR_1_ i non-AN	0.040	0.764	0.447	−0.063–0.144
NLR_1_ i AN	0.253	2.100	**0.039**	0.015–0.489
NLR_2_ i AN	−0.019	−0.159	0.874	−0.258–0.216

**Table 4:** Summary of the random effects of the final model for log(NLR_x_).

Random Effect	SD	σ^2^	95% CI
ID_p_:ID_h_	0.100	0.010	0.000–0.216
ID_p_	0.399	0.159	0.294–0.519
Residuals	0.284	0.081	0.240–0.332

ID_p_—patient ID. ID_h_—hospitalization ID. SD—standard deviation, σ^2^—variance, 95% CI—95% confidence interval.

**Table 5:** Summary of post-hoc tests of differences between groups using the Kenward–Roger method in the model for log(NLR_x_).

Comparison	β	df	t	*p*	95% CI
NLR_1_ and AN vs. NLR_1_ and non-AN	0.213	87.465	1.736	*0.086*	−0.031–0.458
NLR_1_ and AN vs. NLR_2_ and AN	0.273	69.000	2.447	**0.017**	0.050–0.496
NLR_1_ and AN vs. NLR_2_ and non-AN	0.254	87.465	2.065	**0.042**	0.010–0.498
NLR_1_ and non-AN vs. NLR_2_ and AN	0.060	87.465	0.485	0.629	−0.185–0.304
NLR_1_ and non-AN vs. NLR_2_ and non-AN	0.040	69.000	0.764	0.447	−0.065–0.146
NLR_2_ and AN vs. NLR_2_ and non-AN	−0.019	87.465	−0.157	0.876	−0.263–0.225

NLR_1_—neutrophil to lymphocyte ratio value from the first peripheral blood count, NLR_2_—neutrophil to lymphocyte ratio value from the last peripheral blood count, non-AN—non-antipsychotics-naïve, AN—antipsychotics-naïve, 95% CI—95% confidence interval. Statistically significant *p*-values are **bolded**. Statistical trends *p* values are shown in *italics*.

**Table 6:** Summary of the fixed effects of the final model for ΔNLR (model_F_).

Fixed Effect	β	t	*p*	95% CI
Intercept	0.281	1.056	0.301	−0.229–0.790
Age	0.013	2.143	**0.042**	0.002–0.024
Hypothyroidism	0.523	2.695	**0.012**	0.147–0.897
NLR_1_	−0.644	−9.960	**<0.001**	−0.768–−0.768

NLR_1_—neutrophil to lymphocyte ratio value from the first peripheral blood count. 95% CI—95% confidence interval. Statistically significant *p*-values are **bolded**.

**Table 7:** Summary of the fixed effects of the model for ΔNLR with interaction (model_I_).

Fixed Effect	β	t	*p*	95% CI
Intercept	0.284	1.090	0.285	−0.216–0.786
Age	0.013	2.230	**0.035**	0.002–0.024
NLR_1_ with hypothyroidism	−0.343	−3.136	**0.003**	−0.551–−0.134
NLR_1_ without hypothyroidism	−0.656	−10.328	**<0.001**	−0.780–−0.515

NLR_1_—neutrophil to lymphocyte ratio value from the first peripheral blood count. 95% CI—95% confidence interval. Statistically significant *p*-values are **bolded**.

## Data Availability

All data included in the statistical analysis are available on request via the corresponding author.

## Supplementary Materials

The following supporting information can be downloaded at: https://www.mdpi.com/article/10.3390/jcm11010232/s1, Figure S1: Coefficients plot of primary model for log(NLR_x_); Figure S2: Coefficients plot of primary model for ΔNLR (model_P_); Figure S3: Changes in NLR values during hospitalization in the group of antipsychotic-naive patients; Table S1: Incidence of comorbidities among hospitalizations before excluding patients from the research sample and after randomly selecting 300 patient files; Table S2: Summary of the fixed effects of the primary model for NLR_x_; Table S3: Summary of primary model random effects for log(NLR_x_); Table S4: Summary of the fixed effects of the primary model for ΔNLR (model_P_); Table S5: Comparison of the goodness of fit statistics of the primary (model_P_), final (model_F_) and with interaction (model_I_) models for ΔNLR.
