# Supplementary material for: Effect of Antipsychotic Treatment on Neutrophil-to-Lymphocyte Ratio during Hospitalization for Acute Psychosis in the Course of Schizophrenia—A Cross-Sectional Retrospective Study
Source: J Clin Med. 2021 Dec 31;11(1):232. doi: 10.3390/jcm11010232 (PMC8745875; doi:10.3390/jcm11010232)
Supplement: Supplementary file 1 [file jcm-11-00232-s001.zip › jcm-1497856-supplementary.pdf]

SUPPLEMENTARY METERIALS

Table S1. Incidence of comorbidities among hospitalizations before excluding patients from the research sample and after randomly selecting 300 patient files.

Comorbidity	All hospitalizations n_h = 482	Total percent
**Bleeding from the gastrointestinal tract**	1	0,002%
**Peptic ulcer disease**	2	0,004%
Migraine	2	0,004%
**Condition after thyroidectomy**	2	0,004%
**Cholelithiasis**	3	0,006%
**Heart failure**	4	0,008%
**Upper respiratory tract infection**	5	0,010%
**Scabies**	6	0,012%
**Atrial fibrillation**	10	0,021%
**Type II diabetes**	13	0,027%
**Obesity (BMI >30)**	187	0,388%

n_h = 233 have been excluded afterwards. The comorbidities on the basis of which patients were excluded from the study sample are **bolded**.

Table S2. Summary of the fixed effects of the primary model for NLR_x.

Fixed effect	β	t	p	95% CI
Intercept	-0.701	-1.383	0.172	-1.642 – 0.243
BMI	0.024	1.390	0.170	-0.008 – 0.056
Duration of therapy	-0.001	-0.564	0.576	-0.005 – 0.003
Hypertension	-0.225	-1.632	0.109	-0.487 – 0.029
Hypothyroidism	0.239	1.345	0.185	-0.089 – 0.568
Smoking	-0.125	-0.979	0.333	-0.361 – 0.111
Male	0.194	1.465	0.150	-0.050 – 0.438
t_{lag}	0.006	0.233	0.817	-0.042 – 0.053
Age	0.010	1.585	0.121	-0.002 – 0.022
NLR_1 and non-AN	0.040	0.764	0.447	-0.062 – 0.147
NLR_1 and AN	0.308	2.505	**0.014**	0.073 – 0.537
NLR_2 and non-AN	0.035	0.287	0.774	-0.200 – 0.264

BMI – Body-Mass Index, NLR_1 – neutrophil to lymphocyte ratio value from the first peripheral blood count, , NLR_2 – neutrophil to lymphocyte ratio value from the last peripheral blood count, t_{lag} – time from admission to the first blood count, non-AN – non-antipsychotics-naïve, AN – antipsychotics- naïve. 95 % CI – 95% confidence interval. Statistically significant p values are **bolded**.

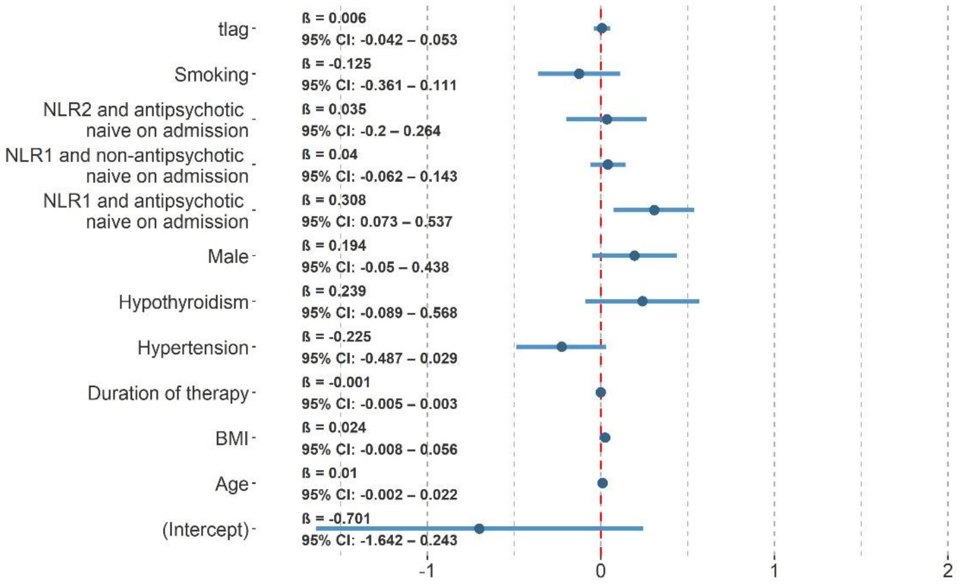

Figure S1. Coefficients plot of primary model for log(NLR_x).

BMI - Body-Mass Index, NLR_1 – neutrophil to lymphocyte ratio value from the first peripheral blood count.

Table S3. Summary of primary model random effects for log(NLRx).

Random effect	SD	σ²	95% CI
ID_p:ID_h	0.049	0.002	0.000 – 0.169
ID_p	0.401	0.161	0.278 – 0.484
Residuals	0.284	0.081	0.244 – 0.321

ID_p – patient ID. ID_h – hospitalization ID. SD – standard deviation, σ² – variance, 95% CI – 95% confidence interval.

Table S4. Summary of the fixed effects of the primary model for ΔNLR ($model_P$).

Fixed effect	β	t	p	95% CI
Intercept	0.385	0.645	0.524	-0.426 – 1.606
BMI	-0.010	-0.495	0.624	-0.053 – 0.018
Duration of therapy	0.002	0.617	0.540	-0.005 – 0.007
Hypertension	-0.245	-1.208	0.233	-0.569 – 0.093
Hypothyroidism	0.524	2.526	**0.018**	0.168 – 0.829
NLR_1	-0.645	-9.338	**<0.001**	-0.741 – -0.510
Smoking	-0.017	-0.116	0.908	-0.266 – 0.215
Male	0.068	0.452	0.656	-0.180 – 0.306
A_{med}	0.167	0.992	0.326	-0.094 – 0.525
t_{lag}	-0.011	-0.290	0.773	-0.075 – 0.056
Age	0.012	1.708	*0.100*	0.000 – 0.022

BMI – Body-Mass Index, NLR_1 – neutrophil to lymphocyte ratio value from the first peripheral blood count, A_{med} – status of antipsychotic medication during the month prior to admission, t_{lag} – time from admission to the first blood count, non-AN – non-antipsychotics-naïve, AN – antipsychotics-naïve. 95 % CI – 95% confidence interval. Statistically significant p values are **bolded**. Trends are shown in *italics*.

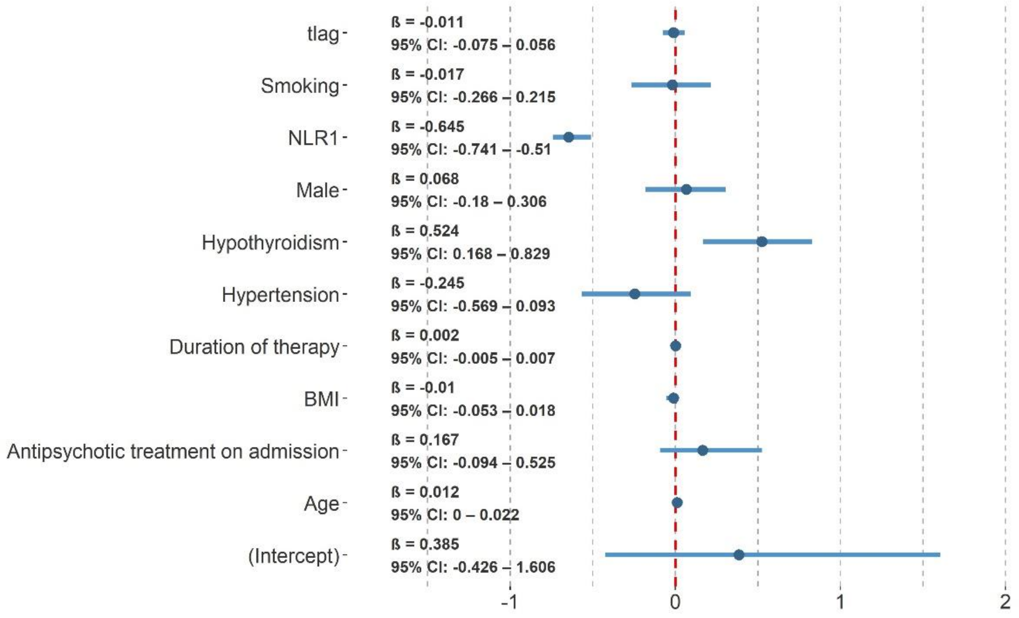

Figure S2. Coefficients plot of primary model for ΔNLR (model$_P$).

BMI – Body-Mass Index, NLR$_1$ – neutrophil to lymphocyte ratio value from the first peripheral blood count, t_{lag} – time from admission to the first blood count.

Table S5. Comparison of the goodness of fit statistics of the primary (model$_P$), final (model$_F$) and with interaction (model$_I$) models for ΔNLR.

Model	AIC	REML
Model$_P$	109.979	131.3
Model$_F$	101.678	107
Model$_I$	98.629	105.4

AIC – Akaike Information Criterion, REML – restricted minimal likelihood.

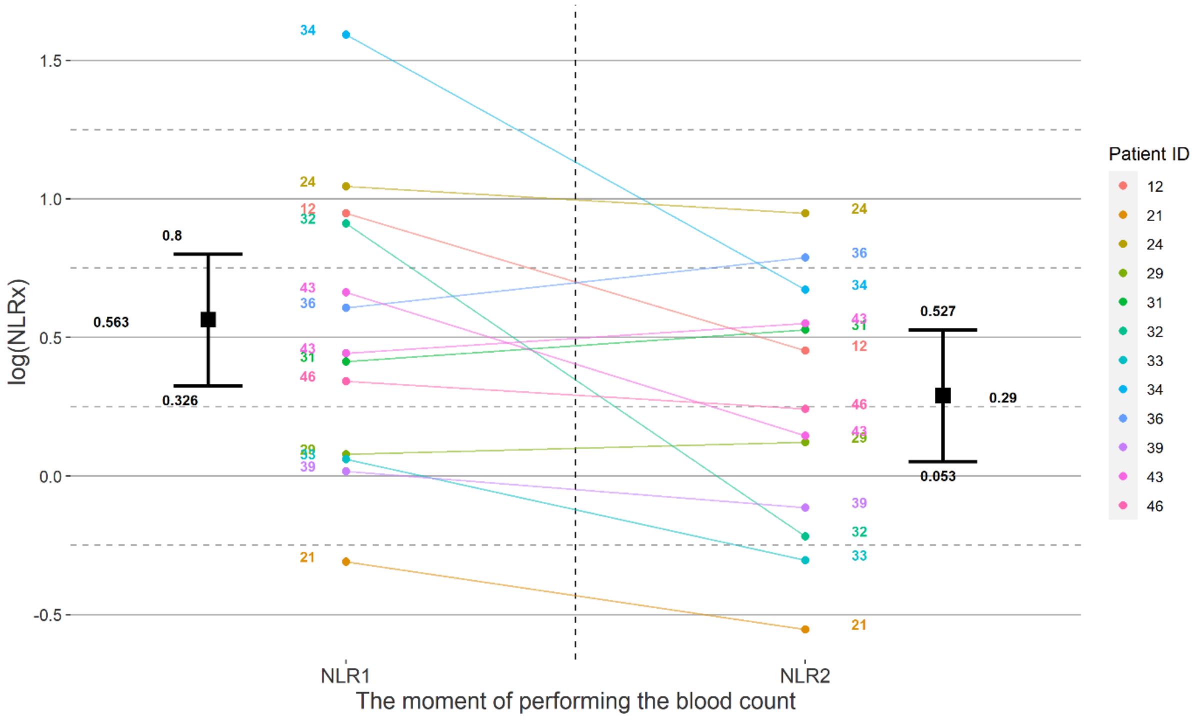

Figure S3. Changes in NLR values during hospitalization in the group of antipsychotic-naive patients.

Points represent NLR values for individual hospitalizations. The differences between the points joined by the lines correspond to the ΔNLR values for the individual hospitalizations. Additionally, the colors indicate the ID of individual patients. Errorbars represent 95% confidence intervals for means. NLR_1 – neutrophil to lymphocyte ratio value from the first peripheral blood count, NLR_2 – neutrophil to lymphocyte ratio value from the first peripheral blood count
